# Novel chicken two-dimensional intestinal model comprising all key epithelial cell types and a mesenchymal sub-layer

**DOI:** 10.1186/s13567-021-01010-z

**Published:** 2021-11-24

**Authors:** Brigid Orr, Kate Sutton, Sonja Christian, Tessa Nash, Helle Niemann, Lone Lind Hansen, Mike J. McGrew, Stina Rikke Jensen, Lonneke Vervelde

**Affiliations:** 1 Division of Infection and Immunity, The Roslin Institute and Royal (Dick) School of Veterinary Studies, University of Edinburgh, Edinburgh, Midlothian UK; 2 Novozymes A/S, Animal Health and Nutrition, 2800 Lyngby, Denmark

**Keywords:** Chicken, Intestinal epithelium, 2D enteroid, Organoid, Barrier integrity, Immune response, Bacteria, Gut model

## Abstract

The intestinal epithelium plays a variety of roles including providing an effective physical barrier and innate immune protection against infection. Two-dimensional models of the intestinal epithelium, 2D enteroids, are a valuable resource to investigate intestinal cell biology and innate immune functions and are suitable for high throughput studies of paracellular transport and epithelial integrity. We have developed a chicken 2D enteroid model that recapitulates all major differentiated cell lineages, including enterocytes, Paneth cells, Goblet cells, enteroendocrine cells and leukocytes, and self-organises into an epithelial and mesenchymal sub-layer. Functional studies demonstrated the 2D enteroids formed a tight cell layer with minimal paracellular flux and a robust epithelial integrity, which was maintained or rescued following damage. The 2D enteroids were also able to demonstrate appropriate innate immune responses following exposure to bacterial endotoxins, from *Salmonella enterica* serotype Typhimurium and *Bacillus subtilis*. Frozen 2D enteroids cells when thawed were comparable to freshly isolated cells. The chicken 2D enteroids provide a useful ex vivo model to study intestinal cell biology and innate immune function, and have potential uses in screening of nutritional supplements, pharmaceuticals, and bioactive compounds.

## Introduction

The small intestine is a highly complex structure with several roles; in addition to nutrient and water absorption from ingested food and secretion of digestive enzymes it acts as physiological barrier, prevents invasion of commensal and pathogenic bacteria in the lumen and regulates the intestinal immune system. An inner folded single layer of endodermal epithelial cells with crypts and villi is exposed to the lumen [1] and beneath the epithelium lies the layered mesenchymal cell architecture of the supportive lamina propria [2]. Homeostasis of the intestine and responses to disease are coordinated by an interplay between the epithelial cells, mesenchymal cells and immune cells through cell–cell contact or soluble factors [1, 2].

To study the intestine ex vivo, three-dimensional (3D) intestinal organoids have been developed in several mammalian species from adult stem cells or isolated crypts and villi including human [3, 4] mouse [4, 5], pig [6], cow [7], and horse [8]. However, the inner lumen of these 3D organoids provide limited access to the apical epithelial surface and experiments often require time-consuming microinjections. Therefore, researchers have generated two-dimensional (2D) polarised models of intestinal monolayers to allow access to the apical side of the cells and measure effects on the epithelial barrier in a standardised way. Successful intestinal monolayers have subsequently been developed in many of these species; human [9, 10], mouse [9, 11, 12], pig [13], cow [14], and cat [15]. For poultry, 2D and 3D ex vivo models of the chicken intestine have been described [16–27]. However, there are currently no chicken intestinal monolayer models that demonstrate expression of all epithelial cell types and form a confluent cell layer with robust epithelial integrity, that can be used to study paracellular permeability and barrier function.

Chickens are precocial birds and by late embryonic development their intestines have formed mature functional epithelial cells in the villi but with only rudimentary crypt structures [28, 29]. The epithelial cells originate from stem cells, but in contrast to mammals, almost all villus cells in poultry are proliferative at hatch with cell mitosis playing an important role in post-hatch hyperplasia [18, 29]. The epithelial layer is made up of absorptive enterocytes, mucin-secreting goblet cells and enteroendocrine cells, Paneth cells and stem cells [25, 30].

Recently, we developed a method for generating 3D outward facing avian enteroids [25], which allows for direct manipulation and access to apical surface of the epithelium, without the need for micro injection. However, due to the 3D structure it is not suitable for automated Trans Epithelial Electrical Resistance (TEER) measurements; TEER is a real-time, non-destructive, label free method to measure epithelial integrity. Existing in vitro chicken 2D enteroids models [18, 24] lacked the ability to form tight confluent cell layers and are limited in their use to study epithelial integrity and permeability. Here, we present an optimised protocol to generate chicken 2D enteroids that contain all major differentiated epithelial cell lineages and a self-organised sub-epithelial mesenchymal layer.

## Materials and methods

### Animals

Experiments were performed using embryonic day (ED18) Hy-Line Brown chickens (*Gallus gallus*) obtained from the National Avian Research Facility, Edinburgh, UK or Lohmann-LSL-LITE Layers from Lohmann, Denmark. Ethical approval was obtained from the Roslin Institute’s and University of Edinburgh Animal Welfare Ethics Review Board. The experiments were performed under the authority of UK Home Office Licenses (PE263A4FA) in accordance within the guidelines and regulations of the UK Home Office “Animals (scientific procedures Act 1986)”.

### Tissue isolation and enteroid culture

Tissue was isolated as previously described [25]. In brief, freshly removed chicken intestine (duodenum, jejunum, ileum and caeca) from ED18 Hy-Line Brown embryos was cut open longitudinally and then transversely into 5 mm pieces. The tissues were washed in Hank’s Buffered Saline Solution [14170595, Thermofisher Scientific, Paisley, UK (TFS)] and digested using collagenase from *Clostridium histolyticum* type IA (0.2 mg/mL, Merck, Gillingham, UK) in DMEM (D5796, TFS) at 37 °C for 50 min with shaking at 200 rpm. Then, after vigorous shaking by hand for 1 min, the release of the villi was checked using an inverted microscope. The larger pieces of tissue settled by gravity and fractions of villi in suspension were collected by filtering the digestion solution over a 70 µM strainer, (CLS231751, Corning, Loughborough, UK), inverting the strainer and washing out the villi with phosphate buffered saline (PBS) into a petri dish. Then, the fractions containing the villi were pooled and pelleted at 100 × *g* for 4 min. Aliquots of each fraction were checked for purity using an inverted microscope. Cell pellets were resuspended in accutase (A1110501, TFS) and incubated for 5 min at 37 °C, mechanically disrupted by pipetting and further incubated for 5 min at 37 °C. Successful cell dissociation of epithelial and mesenchymal cells was checked and accutase activity was diluted in the cell suspension with 2 volumes of Advanced DMEM/F12 (12634010, TFS) and filtered twice through a 40 µM strainer (CLS431750, Corning) to eliminate cell aggregates. Cells were collected by centrifugation at 200 × *g* for 4 min. Cells were resuspended in Seeding Media (Table 1) and incubated for 3 h at 37 °C, 5% CO_2_ in a 12-well plate to allow for preferential mesenchymal cell adherence. The single cell mix of epithelial cells and non-adherent mesenchymal cells was then used to seed the 2D enteroids (day 0) on 96-well plates or apical inserts of 24-well transwells (CLS-3396, Corning), coated with growth factor reduced Matrigel (354230, Corning; protein concentration ~ 10 mg/mL 1:50 diluted in Basal Media), which had previously been allowed to polymerise for 1 h at 37 °C. Prior to seeding of the cells, excess Matrigel was removed from the wells and immediately replaced with Seeding media (Table 1). Cells were seeded at 1 × 10^5^ using freshly isolated cells or 1–2 × 10^5^ cells from the frozen cell stocks in Seeding Media (Table 1). For transwells, a total of 200 µL of media/well was added to the apical compartment and 500 µL of media was added to basal compartment. After 24 h, cells were washed in Basal Media, and cultured with Maintenance Media, which was replaced every 2 days. Cells were incubated at 37 °C, 5% CO_2_.

**Table 1:** **List of reagents used to prepare cell culture media.**

Reagent	Concentration	Cat. No	Supplier
Basal media			
Advanced DMEM/F12	1 ×	12634010	ThermoFisher scientific (TFS)
l-glutamine	2 mM	25030024	TFS
HEPES	10 mM	15630080	TFS
Penicillin/streptomycin	50 U/mL	TFS	15140122
Seeding media			
Basal media	1 ×		
B27 supplement	1 ×	17504044	TFS
N2 supplement	1 ×	17502048	TFS
EGF (human)	100 ng/mL	PHG6045	TFS
LDN 193189	100 nM	BV-2092-5	Cambridge bioscience
R-Spondin (human)	100 ng/mL	4645-RS	R&D systems
CHIR 99021	10 µM	S1263-SEL	Stratech scientific
Y27632	10 µM	72304	Stem cell technologies
Maintenance media			
Basal media	1 ×		
B27 supplement	1 ×		
N2 supplement	1 ×		
EGF (human)	100 ng/mL		
Noggin (human)	50 ng/mL	6057_NG_025	R&D systems
R-Spondin (human)	100 ng/mL		

Cell culture images were captured on a Nikon TE300 microscope camera and Zen (Black) software (Zeiss, Jena, Germany).

### Immunofluorescence

Confluent cell layers in 96-well plates or 24-well transwell membrane inserts were gently washed with PBS, fixed in 4% paraformaldehyde (PFA; 15670799, TFS) for 10 or 20 min respectively and washed again with PBS. Cells in 96-well plates were permeabilised with 0.1% saponin (84510, Sigma, Gillingham, UK)/0.5% bovine serum albumin (BSA; A2153, Sigma)/PBS or 0.1% Triton-X100 (Triton-X, Sigma)/0.5% BSA/PBS for 10 min at RT and blocked with 5% goat serum in permeabilisation buffer for 1 h at RT. Cells were incubated overnight at 4 °C with primary antibodies diluted in blocking buffer, followed by species-specific secondary antibodies (2 mg/mL) or isotype controls diluted in permeabilisation buffer and incubated for 1 h at RT.

Cells on transwell membrane inserts were permeabilised with 0.5% Triton X/PBS for 10 min at RT and blocked with blocking buffer 10% goat serum/0.1% Triton-X/PBS. Primary antibodies were diluted in 0.1% Triton-X/2% BSA/PBS and incubated with cells for 1 h at RT. Antibodies were visualised with species and isotype specific secondary antibodies and phalloidin rhodamine diluted in in 0.1% Triton-X/2% BSA/PBS and incubated for 15 min at RT. Primary and secondary antibodies are listed in Table 2.

**Table 2:** **List of primary and secondary antibodies used for immunocytochemistry.**

Antibody	Antigen	Isotype	Cat no.	Supplier	Working dilution
Mouse anti-chicken CD45 (clone AV53)	CD45	IgG1	n/a	Institute for Animal Health, UK	1:100
Rabbit anti bovine SP-1 Chromogranin A	Chromogranin A	IgG	20085	Immunostar	1:750
Mouse anti-human E-cadherin (clone 36)	E-cadherin	IgG2a	36/Ecadherin	BD Biosciences	1:50
Phalloidin Rhodamine	F-actin		R415	Invitrogen	1:100
Rabbit anti-chicken Lysozyme (polyclonal)	Lysozyme C	Poly IgG	Ab391	Abcam	1:50
Rabbit anti-human Muc5AC (clone 45M1)	Muc5AC	IgG1	Ab212636	Abcam	1:50
Mouse anti-chicken Villin (clone 1D2C3)	Villin	IgG1	Sc-58897	Santa Cruz	1:50
Mouse anti-pig Vimentin (clone V9)	Vimentin	IgG1	Ab8069	Abcam	1:50
Rabbit anti-human ZO1 (polyclonal)	ZO1	IgG	Ab216880	Abcam	1:100
Mouse anti-human ZO1 (clone A12)	ZO1	IgG1	33-9100	Invitrogen	1:100
Goat anti mouse Alexafluor^®^ 488	Mouse IgG1	IgG1	A-21121	Invitrogen	1:200
Goat anti mouse Alexafluor^®^ 488	Mouse IgG	IgG	A-11001	Invitrogen	1:200
Goat anti mouse Alexafluor^®^ 546	Mouse IgG2a	IgG2a	A-21133	Invitrogen	1:200
Goat anti rabbit Alexafluor^®^ 568	Rabbit IgG	IgG	A-11036	Invitrogen	1:200
Goat anti rabbit Alexafluor^®^ 647	Rabbit IgG	IgG	A-21244	Invitrogen	1:200

For immunolocalisation of the subepithelial basal mesenchymal cells, prior to fixation, cells were incubated in 1 mM EDTA/0.5% Triton-X/PBS for 20 min and flushed with PBS for removal of apical epithelial cells using a pastette. Control samples were incubated with isotype controls or secondary antibodies only. Nuclei were visualised with 4′,6-diamidino-2-phenylindole (D9542, DAPI; Sigma). Transwell membrane inserts were removed for mounting in ProLong Diamond Antifade Mountant (P36965, TFS).

Fluorescent images on cell culture plates and thawed mesenchymal cell layer were captured using an Axiovert 25 microscope with Axiocam 503 colour camera (Zeiss) and Zen (Black) software (Zeiss). Mounted transwells were imaged using an Olympus 3000 inverted laser scanning microscopy with the PLAPON 60XOSC objective lens. Software acquisition and analysis was performed using FV31S-SW and cellSens software (Olympus, Tokyo, Japan).

### Measurement of permeability of 2D enteroids

To measure permeability to small molecules, 2D enteroids on matrigel coated transwells were apically treated with 0.5 mg/mL 4 kDa FITC-dextran (FD40S, Sigma) and at 0.5, 1, 2, 3, 4 h post application, 50 µL of basal media was removed, and fluorescence measured on a Clariostar plate reader (BMG Labtech, Aylesbury, UK) with 490 nm excitation and 530 nm emission. At each time point, fluorescence measurements were taken in duplicate from three wells containing cells, two coated wells with no cells and one well with media only. After each measurement, the media samples were returned to their original compartments. Fluorescence was normalised against the measurement of media only (no cells and no FITC-dextran) across all time points.

### Trans epithelial electrical resistance (TEER) of 2D enteroids

TEER was measured using an epithelial voltohmmeter (EVOM2, World Precision Instruments, Hitchin, UK) and chopstick electrode (STX2). In all experiments, the TEER of coated wells without cells was ~ 16–30 Ω·cm^2^. The final TEER value was calculated from the following equation, TEER = (R_cell layer_ − R_blank_) × A. R_cell layer_ is the resistance of the cell layer with the coated filter membrane; R_blank_ is the resistance of the coated filter membrane without cells, and A is the surface area of the membrane (0.33 cm^2^). To compare between experiments, all wells were normalised to controls in the same experiment. Cells in 24-well transwells were cultured with 200 µL apical and 500 µL basal media. Media was replaced in the apical and basal compartments every 1–2 days. TEER readings were performed twice per well for duplicate wells from day 3, when a confluent 2D enteroid with maximum TEER was formed.

### Manipulation of 2D enteroids with treatments or bacterial endotoxins

For measurement of TEER, treatments were added to Maintenance Media or Low Calcium Media (LCM) in the apical and basal compartments in Matrigel-coated transwells. Treatments were added from day 3, when a confluent cell layer had formed and replaced every 2 days with Maintenance Media. On day 3 the cell layers were treated with 3-, 5-, or 7 mM sodium butyrate (NaB; 303410, Sigma) or with 1.2–2 mM calcium chloride (99609, Sigma). Low calcium media was prepared by combining DMEM (no Ca^2^^+^; 21068028, TFS) with Advanced DMEM/F12 (TFS) in appropriate ratios to make LCM containing 0.26 mM calcium.

To measure the effect of pathogen challenge, the 2D enteroids were treated with heat-inactivated avian pathogenic *Escherichia coli* O1 serotype 01:K1:H7 (HiAPEC) (multiplicity of infection; MOI 1, 10, 100), Lipolysaccharide (LPS) from *Salmonella enterica* serotype Enteridis (*S.* Enteridis; L7770), *Salmonella enterica* serotype Typhimurium (*S.* Typhimurium; L6143), *Escherichia coli* (*E. coli* 055:B5; L6529) or Lipoteichoic acid (LTA; L3265, all from Sigma) at 0.1, 1 or 10 μg/mL. APEC O1-eGFP was grown and eGFP expression confirmed as previously described [31]. HiAPEC was prepared by calculation of CFU/mL and subsequent heat inactivation at 56 °C for 24 h; loss of replication was checked on agar plates in the absence of antibiotics.

For qRT-PCR analysis, small intestines were pooled from five embryos and enteroids were prepared, as described earlier. Cells were seeded at 3 × 10^6^ cells per well on non-coated 24-well plates in Seeding Media that was replaced with Maintenance Media after 24 h and on day 3 and 5. On day 5 of culture, 2D enteroids were treated apically with or without 1 or 10 µg/mL of LPS from *S. typhimurium* or LTA from *Bacillus subtilis* (*B. subtilis;* Sigma) for 6 h at 37 °C, 5% CO_2_. Cells were lysed with RLT buffer (Qiagen, Gillingham, UK) and stored at −20 °C until use.

### Quantitative real-time polymerase chain reaction

Total RNA was extracted using an RNeasy Plus Mini Kit with a gDNA column eliminator (74134, Qiagen) according to manufacturer’s instructions. Reverse transcription was performed using the Superscript III First Strand Synthesis System (18080051, TFS) according to manufacturer’s instructions using random hexamers and oligo (dT)18 and 1 µg of total RNA using G-STORM GS-1 thermal cycler (Gene Technologies, TFS). The cDNA samples were stored at −20 °C until use. To measure mRNA levels, 1:5 dilution of cDNA was mixed with 10 µL of ABI TaqMan Gene Expression Master Mix (4369016, Applied Biosystems, TFS), 1 µL of 20X EvaGreen (31000, Biotum, VWR-Bie & Berntsen, Lutterworth, UK) and specific primer pairs (forward and reverse) at a final concentration of 1.15 μM for interleukin 6 (IL-6, F:GCTCGCCGGCTTCGA, R:GGTAGGTCTGAAAGGCGAACAG, Acc. No. AJ309540.1), IL-8L2 (CXCLi2, F:GCCCTCCTCCTGGTTTCAG, R:TGGCACCGCAGCTCATT, Acc. No. FNM_205498.1), α smooth muscle actin (αSMA, F: TCTGGGCGTACTACAGGGAT, R: GCATGATGGCATGTGGCAAA, Acc. No. NM_001031229.1), Desmin (F:CGAGGAGAACCGGATCAGCA, R: TTCTTGGTGTGCACCTCGG Acc. No. XM_015290092.3) and housekeeping genes, glyceraldehyde phosphate dehydrogenase (GAPDH, F:GAAGGCTGGGGCTCATCTG, R:CAGTTGGTGGTGCACGATG, Acc. No. AF047874) and ribosomal 28S (r28S, F:GGCGAAGCCAGAGGAAACT, R: GACGACCGATTTGCACGTC, Acc. No. FM165415), as previously described [32]. Reactions were performed in triplicate and no template controls without cDNA were included to detect any potential non-specific amplification. Efficiency of each reaction was calculated based on serial tenfold dilutions of calibration sample. The qPCR reaction was carried out at 50 °C for 2 min, 95 °C for 10 min followed by 40 cycles at 95 °C for 15 s and 60 °C for 1 min. Data normalisation was based on the geometric mean of two reference genes, r28S and GAPDH. Relative quantities of IL-6 and CXCLi2 were calculated compared to the untreated control group. The expression of Lgr5, αSMA and desmin mRNA in 2D cultures was calculated using the corrected 40-Ct method as previously described [33].

### Cryopreservation of enteroids

To cryopreserve the isolated cells, non-adherent cells containing epithelial cells and some mesenchymal cells were pooled from wells after the adherence step and pelleted at 200 × *g* for 4 min at RT. Media was removed and cells were resuspended in Cryostor CS10 cryopreservation medium (7930, Stem Cell Technologies, SCT, Cambridge, UK) containing 10 mM Y27632 (72304, SCT), at 1 × 10^6^ cells/mL. Cryovials were stored overnight in a Mr Frosty freezing container (5100-0001, TFS) at −80 °C, and then transferred to liquid nitrogen for long-term storage.

To resuscitate the cells, the cryovials were thawed briefly in a water bath at 37 °C. The cell suspension was transferred to 5× volume of DMEM/F12 and pelleted by centrifugation at 200 × *g* for 4 min at RT and cultured as previously described. Cells were seeded at a concentration of 1–2 × 10^5^ cells/well in 24-well transwells and CHIR99021 (S1263-SEL, Stratech Scientific, Ely, UK) was added in the Seeding or Maintenance Media until day 1 or day 3, respectively.

### Statistical analysis

Statistical analysis was performed using GraphPad Prism 8.00 (GraphPad, San Diego, USA). All data was analysed for normality and TEER data was analysed by One-Way ANOVA with post-hoc Dunnett multiple comparison. qRT-PCR data was analysed by non-parametric Kruskal–Wallis test adjusted for post-hoc analysis. The probability level for significance was taken as *P* ≤ 0.05.

## Results

### Establishment of 2D enteroids culture system

**Figure 1:**
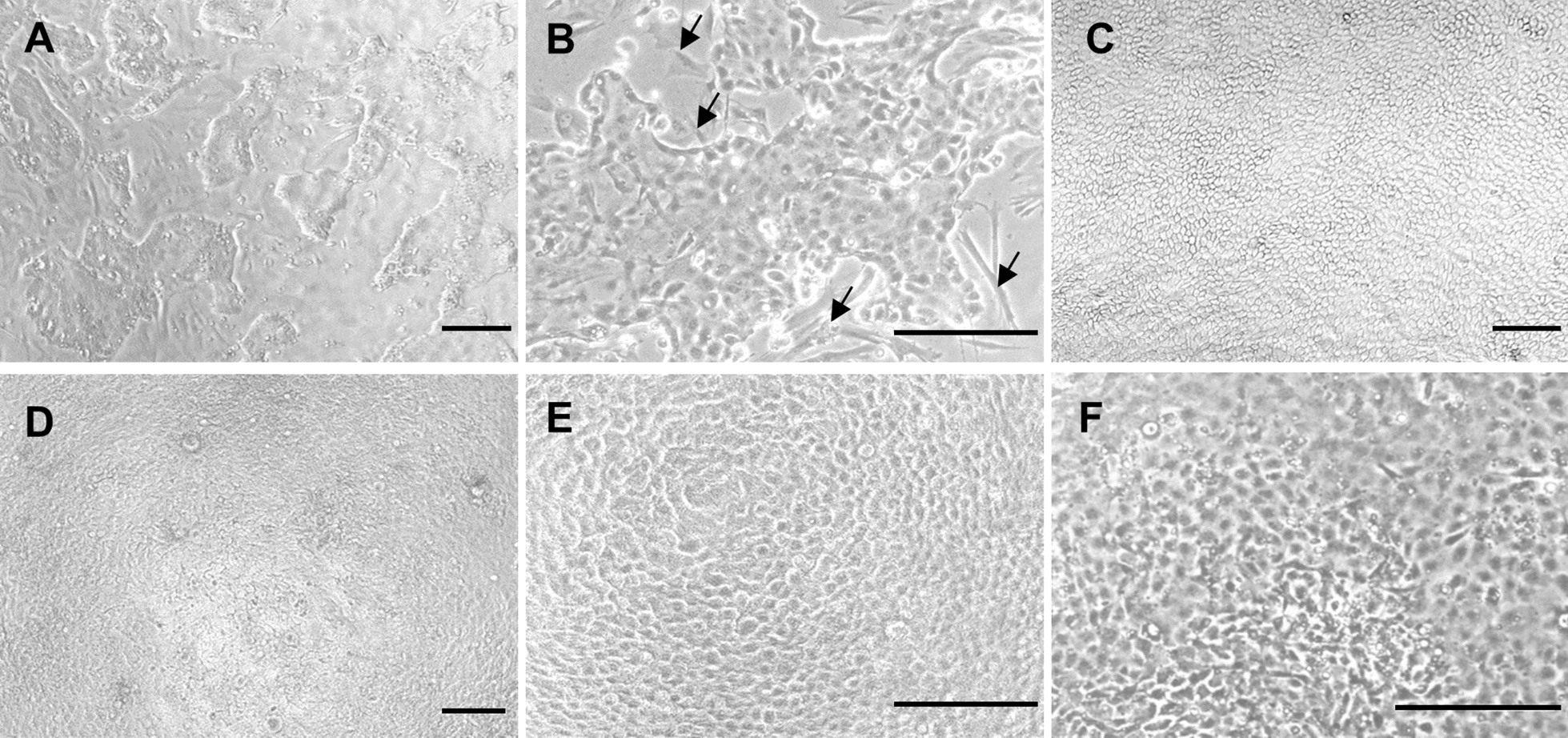
**Growth and formation of 2D enteroids.** 2D enteroid grown on Matrigel-coated cell culture plastic. **A** Bright field image of epithelial cells forming islands on day 1 of culture. **B** Fibroblast-like cells seen around the edge of the epithelial cell islands (arrows). **C** A confluent polygonal epithelial cell layer on day 3 and on **D** day 6 of cell culture. **E** Higher magnification of 2D enteroid on day 6 of culture. **F** Bright field image of 2D enteroid grown on an uncoated cell culture plastic. Representative images of 5–6 independent experiments. Scale bars: 100 µm.

To establish a chicken 2D enteroid culture system, we plated dissociated single cells from villi isolated from the intestine of ED18 embryos in a cell culture medium containing the growth factors, EGF, Noggin, R-spondin, CHIR99021 and ROCK inhibitor. We observed the initial formation of epithelial islands after 24 h (Figures 1A and B). The islands expanded to generate a confluent layer of epithelial cells by 3 days (Figure 1C), with the characteristic polygonal shape and cobblestone appearance found in 2D enteroids, also present at 6 days of culture (Figures 1D and E). Cells were most successfully grown on Matrigel coated wells but could also be grown to form confluent layers on uncoated cell culture plastic (Figure 1F).

### Identification of multi-cell lineages in 2D cultures

**Figure 2:**
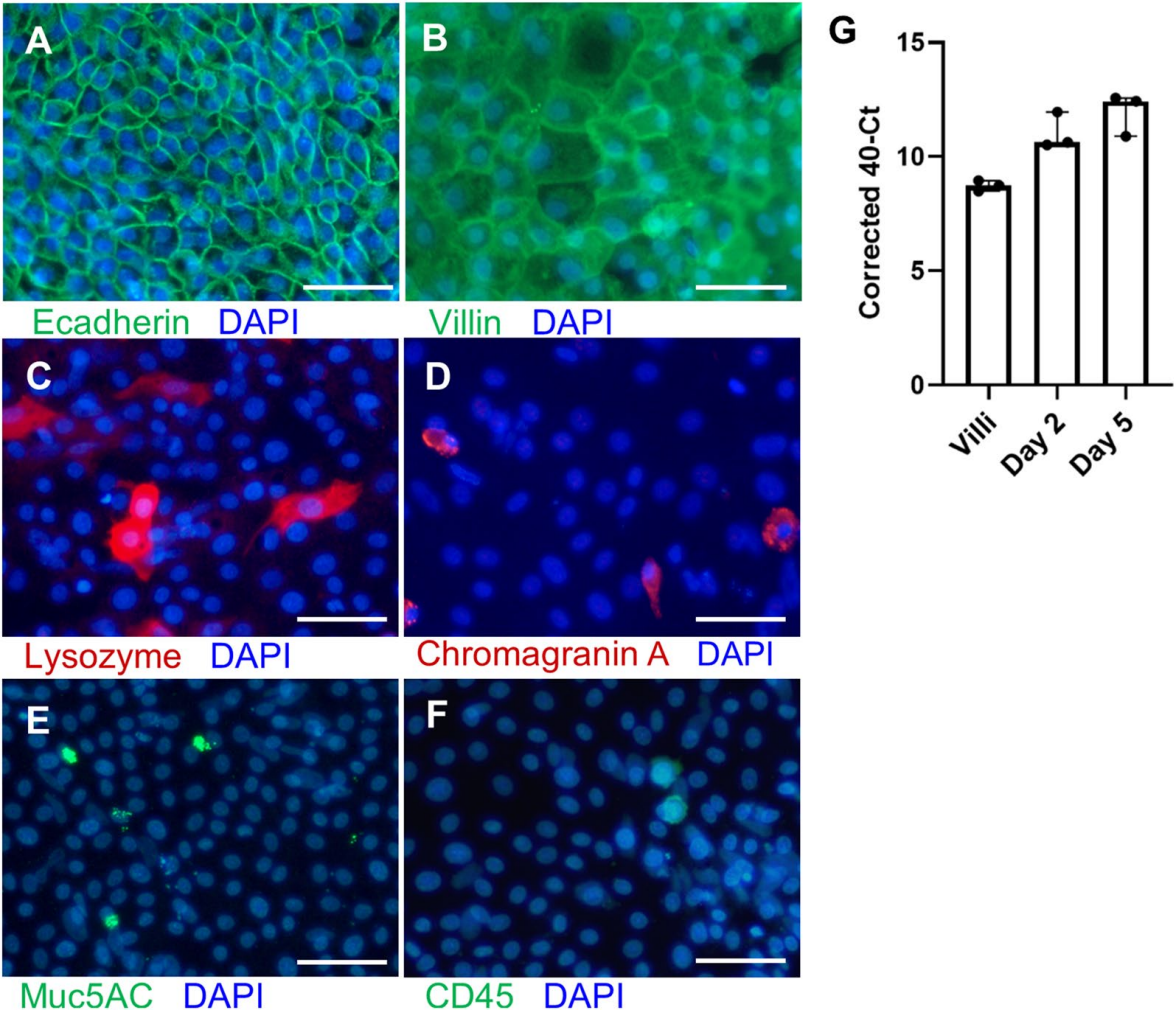
**Cellular characterisation of the apical layer of 2D enteroids**. Immunolocalisation of (**A**–**F**) apical epithelial cell types in 2D enteroids grown on Matrigel coated cell culture plastic at day 6 of culture. All cells are counterstained with DAPI (blue). The cells are stained for **A** E-cadherin (epithelial cells with adherens junctions, green) and vimentin (red), but vimentin expression is not evident in the sub-epithelial layer after mild cell permeabilisation with saponin, **B** villin (enterocytes, green), **C** Lysozyme (Paneth cells, red), **D** Chromogranin A (enteroendocrine cells, red), **E** Muc5AC (Goblet cells, green), and **F** CD45^+^ (leukocytes, green). Representative images from 3–4 independent experiments. Scale bars: 50 µm. **G** mRNA expression of the stem cell marker Lgr5 in freshly isolated villi, at day 2 and 5 of culture, grown on uncoated cell culture plastic. Data represents the median and 95% CI of three independent experiments.

To further characterise these 2D enteroids, immunofluorescent imaging was performed. All major differentiated epithelial cell lineages were present in the cultures corresponding to those present in the intestine. Epithelial cells were identified by E-cadherin expression at the cell surface, marking adherens junctions, and cytoplasmic villin staining indicated the presence of mature absorptive enterocytes. Paneth cells were identified by lysozyme expression and Goblet cells by Muc5AC staining. Chromogranin A expression indicated the presence of entero-endocrine cells and CD45^+^ leukocytes identified intra-epithelial leukocytes in the 2D enteroids (Figures 2A–F). The presence of stem cells was confirmed by Lgr5 mRNA expression (Figure 2G).

**Figure 3:**
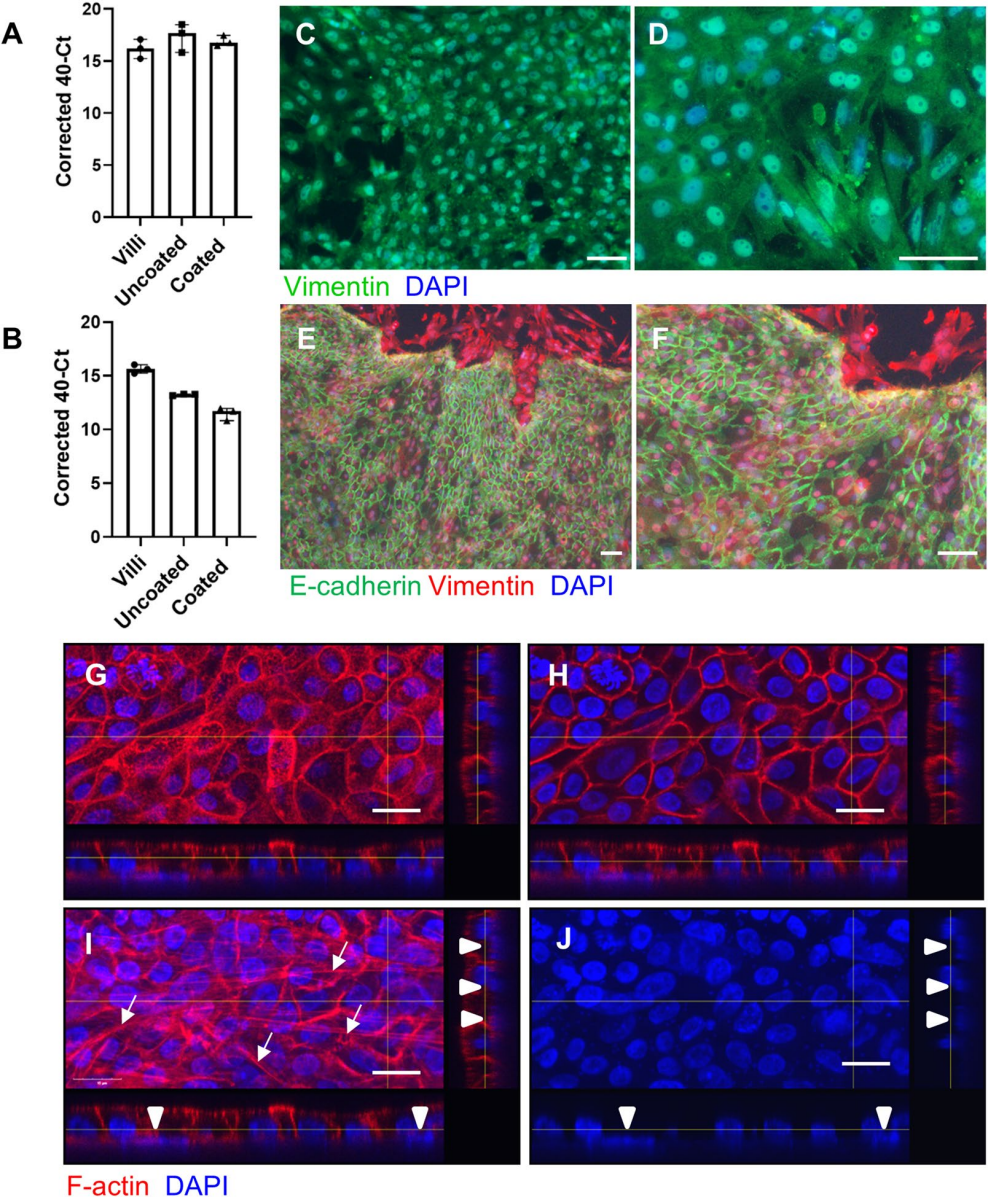
**Cellular characterisation of the basal layer of 2D enteroids.** mRNA expression of **A** α-smooth muscle actin and **B** desmin in freshly isolated villi and in 2D enteroids grown on uncoated and Matrigel coated cell culture plastic at day 5 of culture. Data is represented as the mean of three independent experiments and 95% CI. Immunolocalisation of basal mesenchymal cell types in 2D enteroids grown on Matrigel coated cell culture plastic (**C**–**F**) or transwells (**G**–**I**). After removal of the apical cells, the cells are stained for **C**, **D** vimentin (mesenchyme, green). **E**, **F** To demonstrate the subepithelial layer, the cells are permeabilised with Triton X100 on day 2 and double stained for E-cadherin (epithelial cells with adherens junctions, green) and vimentin (mesenchyme, red). Vimentin expression is evident in the sub-epithelial layer. Images from 3 independent experiments. All cells are counterstained with DAPI (nuclei, blue). Scale bars: 50 µm. (G-J) Immunolocalisation of F-actin (red) and DAPI (nuclei, blue) in 2D enteroids grown on Matrigel coated transwells at day 4 of culture. Z- stack of **G** Apical, **H** Middle and **I**, **J** Basal slices. **I**, **J** DAPI stained nuclei in the basal slices demonstrate the presence of nuclei in the subepithelial layer (white arrowheads), whereas long F-actin filaments from mesenchymal cells (**I**) cross the basal layer slice (white arrows). The lines on the main photo indicate the image area represented in the transverse slices at the bottom and side of each photo. Representative images of 1 experiment. Scale bars: 15 µm.

Further characterisation indicated that the cultures consisted of two cell layers with a sub-epithelial cell layer of mesenchymal cells. These cells expressed α-smooth muscle actin and desmin mRNA (Figures 3A and B). The sub-epithelial layer was revealed by staining of vimentin after removal of the apical cells, (Figures 3C and D) and by co-staining of vimentin and E-cadherin (Figures 3E and F). A Z-stack of immunolocalisation of F-actin and nuclei (DAPI) in the 2D enteroids showed basal nuclei and long F-actin filaments characteristic of mesenchymal cells in the basal slices (Figures 3G–J). The data also confirms the epithelial layer is polarised, with the apical side facing the media, while the basolateral region contained a layer of mesenchymal cells. These results show that we have generated an ex vivo 2D enteroid intestinal culture that recapitulates all differentiated epithelial cells and a sub-epithelial mesenchymal cell layer with similarity to the chicken intestine.

### Intestinal epithelial cells form a tight cell layer on transwells

**Figure 4:**
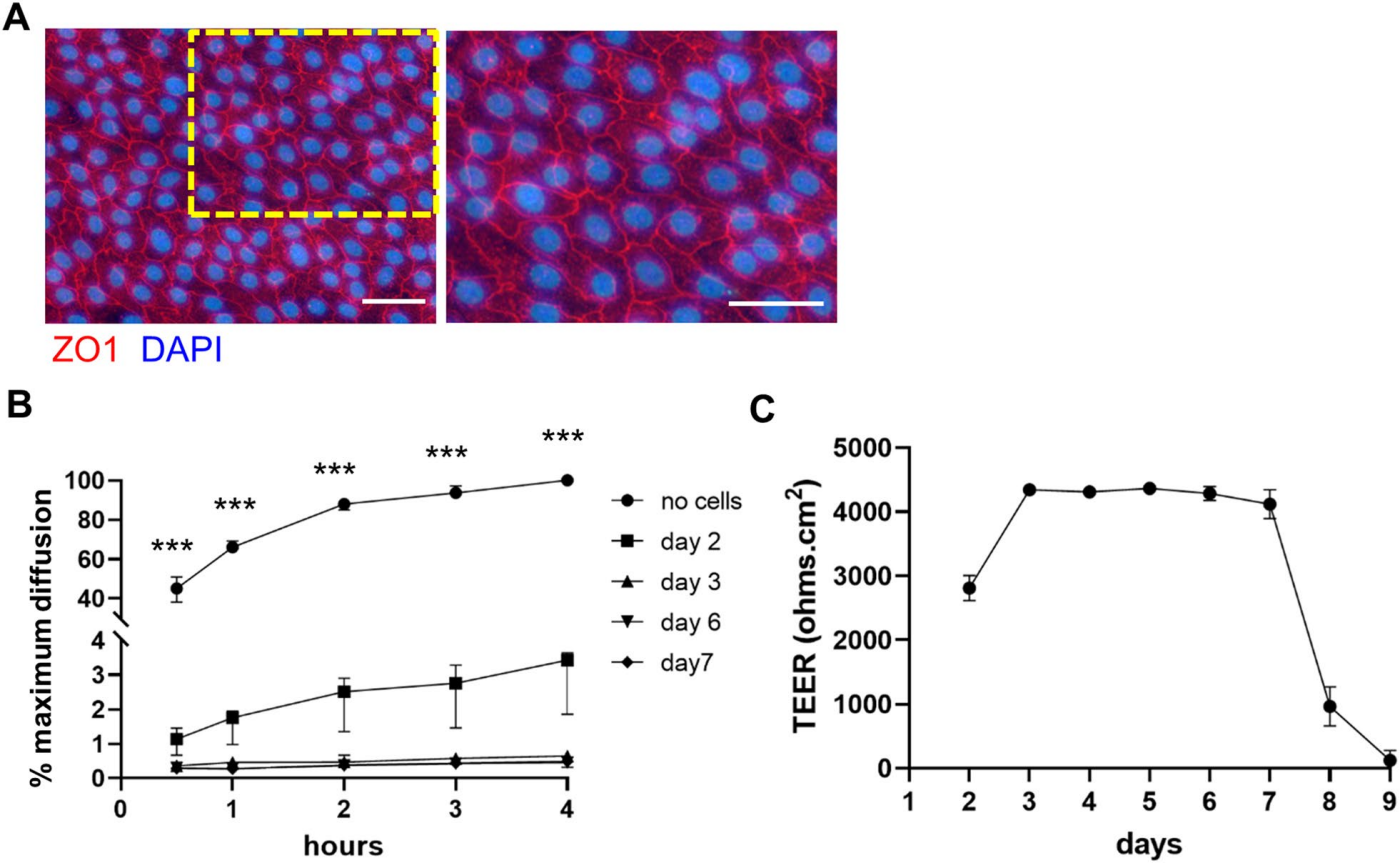
**Tight junction formation of 2D enteroids.** 2D enteroids grown on Matrigel coated **A** cell culture plastic or **B**, **C** transwells. Cells are stained after 3 days in culture for ZO1 (red) expression with a low power image (**A**) and inset magnified. Nuclei are stained with DAPI (blue). Representative images of four independent experiments. Scale bar: 30 µM. **B** Paracellular transport measured by diffusion of 4 kDa FITC-dextran across the 2D enteroids from the apical to basal compartment in Matrigel coated transwells. Measurements were taken at 0.5, 1, 2, 3, 4 h post-application of FITC-dextran to the apical chamber. To allow for normalisation across all time points, fluorescence measurements included a sample of cell culture media only. Data is converted to percentage maximum diffusion, measured in wells with Matrigel coated inserts and no cells. ***Represents statistically significant difference between Matrigel coated wells with cells and with no cells. Data is the median of four independent experiments and 95% CI. (C) Measurement of trans-epithelial electrical resistance (TEER) reaching maximum TEER at day 3 and maintained until day 7 of culture. Data is represented as median of four independent experiments and 95% CI.

We next tested the ability of the 2D enteroids to form a function barrier, an essential parameter for a physiological relevant intestinal model. Expression of the tight junction protein Zona Occludens 1 (ZO1) was detected in the epithelial cells connecting the cytoskeleton of adjacent cells (Figure 4A). We measured paracellular permeability of the 2D enteroids to small molecules by diffusion of 4 kDa FITC-dextran in a transwell system. Measurement of fluorescence in the basal compartment demonstrated minimal transport was observed from day 3 of culture (Figure 4B), whereas in control wells with no cells, significantly more movement of FITC-dextran into the basal compartment was detected. Using the transwell system, we measured TEER and found that from day 3 of culture the 2D enteroids reproducibly gave a consistent TEER value of 4000–4500 Ω·cm^2^ (Figure 4C) which lasted to day 7. The drop in TEER at day 8 was not caused by cell death, but due to the apical and basal layer detaching from the edge of the transwell or less frequently by the appearance of holes in the apical layer. At day 2 of culture when the 2D enteroid was not completely developed, some level of paracellular permeability was observed which was consistent with the sub-optimal TEER measurement at this timepoint.

### Manipulation of epithelial integrity of the 2D enteroids

**Figure 5:**
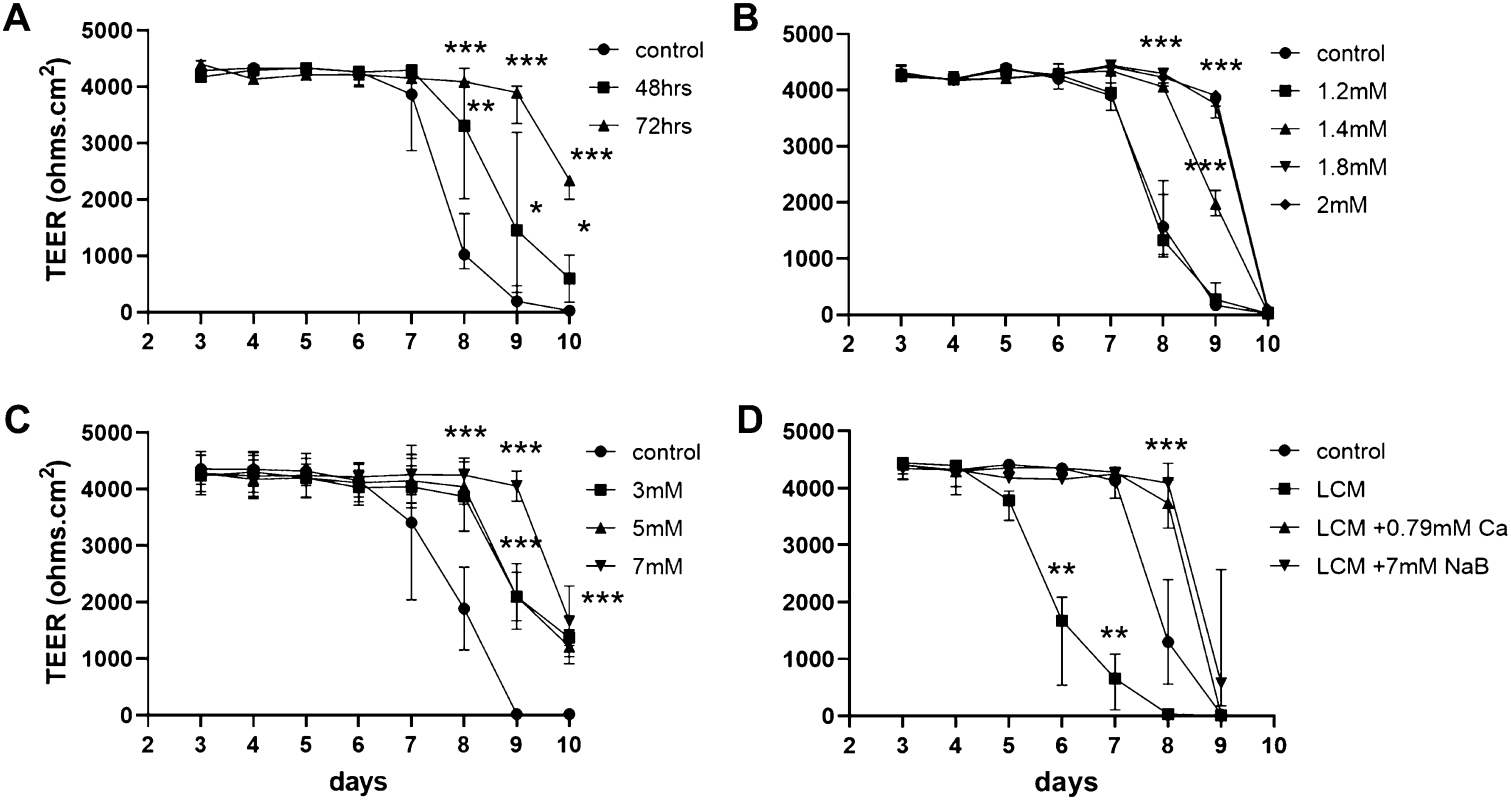
**Enhancement of intestinal epithelial integrity.** 2D enteroids grown on Matrigel coated transwells. The duration of the maximum TEER of 2D enteroids grown can be extended by **A** addition of CHIR99021 (10 mM) in the culture media from initial seeding for longer than 24 h (control, 24 h), **B** increasing Ca^2+^ concentration from control concentration 1.05 mM to 2 mM by addition of calcium chloride (duplicate wells) or by **C** addition of sodium butyrate (NaB; 3, 5, 7 mM; duplicate wells). **D** NaB and Ca^2+^ rescue epithelial integrity of a leaky gut model of 2D enteroids grown in low calcium media (LCM with 0.26 mM Ca^2^^+^). The barrier integrity could be rescued by restoring Ca^2^^+^ concentration to the control concentration (1.05 mM Ca^2^^+^) or by adding NaB (7 mM) from day 3 of culture. All additions were made every second day, from day 3 of culture, to the apical and basal compartments of the transwell. Statistical significance compared to control wells are *P < 0.05, **0.05 < *P* > 0.001, ***P < 0.001. Data is presented as the median of 3–5 independent experiments and 95% CI.

To investigate the functional capability of the 2D enteroids we first tested several factors known to modulate and maintain epithelial integrity. First, we altered the duration of Wnt activator CHIR99021 in the Seeding Media for 24 h (control) and then to the Maintenance Media at 48 h or 72 h, and the prolonged presence significantly increased the duration of the maximum TEER from day 7 up to day 9 of culture (Figure 5A).

Second, we investigated the effect of increasing the Ca^2+^ concentration on 2D enteroid integrity. The Basal Media Advanced/DMEM F12 (control) has a relatively low concentration of Ca^2+^ (1.05 mM) in comparison to the 1.8 mM Ca^2+^ physiological concentration of many cell culture media. The effect of increased Ca^2+^ concentrations in the Maintenance Media resulted in prolonged maximum TEER levels in a concentration-dependent manner (Figure 5B).

Third, we investigated the effect of sodium butyrate (NaB) on 2D enteroid epithelial integrity. NaB is known to regulate tight junction assembly of the gut epithelial layer. NaB prolonged the duration of the maximum TEER in a concentration-dependent manner; addition of 7 mM NaB extended the maximum TEER beyond control, day 7, up to day 9 of culture (Figure 5C). Thus, the 2D enteroids demonstrated the functional ability to respond to Wnt activation, increased Ca^2+^ concentration and NaB, to support and protect epithelial junction integrity during prolonged cell culture.

Following the observed positive effect of NaB on our 2D enteroid model of a healthy gut, we investigated the ability of NaB to rescue or restore epithelial integrity to a leaky gut with reduced epithelial integrity. A leaky gut model was generated by manipulation of the 2D enteroids; switching to LCM media (0.26 mM Ca^2+^ concentration) from day 3 of culture, when confluency and maximum TEER was established resulted in a drop in TEER. Restoration of the Ca^2+^ concentration to control levels (1.05 mM) by addition of CaCl_2_ restored the maximum TEER values (Figure 5D). Similarly, NaB added to 2D enteroids cultured in LCM media was also able to restore epithelial integrity. In LCM media, the maximum TEER measured in the 2D enteroids declined from day 5 of culture, but in the presence of NaB the maximum TEER was maintained until day 8 (Figure 5D).

### Stimulation of 2D enteroids with bacterial endotoxins

**Figure 6:**
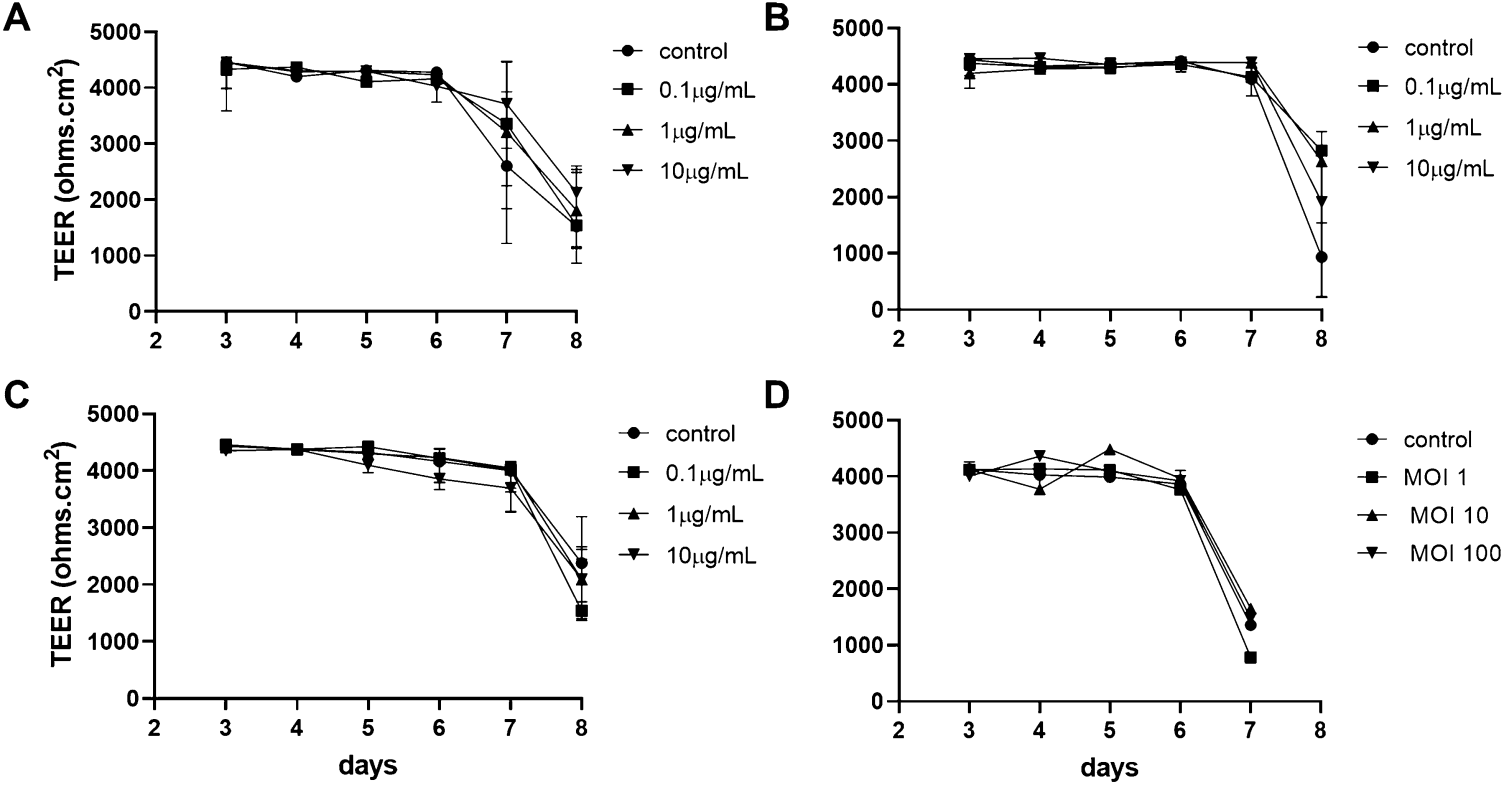
**Bacterial components have no effect on intestinal epithelial integrity.** 2D enteroids grown on Matrigel coated transwells. LPS from **A** *S. enterica* serotype Typhimurium, **B** *E. coli* O55:B5, **C** *S. enterica* serotype Enteritidis (0, 0.1, 1 and 10 μg/mL) and **D** Heat inactivated Avian Pathogenic *E. coli* (MOI 0, 10, 10, 100) were added from day 3 of culture to the apical and basal compartments. TEER measurements were analysed from day 3 to day 7 or 8 of culture. Data is represented as the mean of 3 independent experiments and 95% CI.

**Figure 7:**
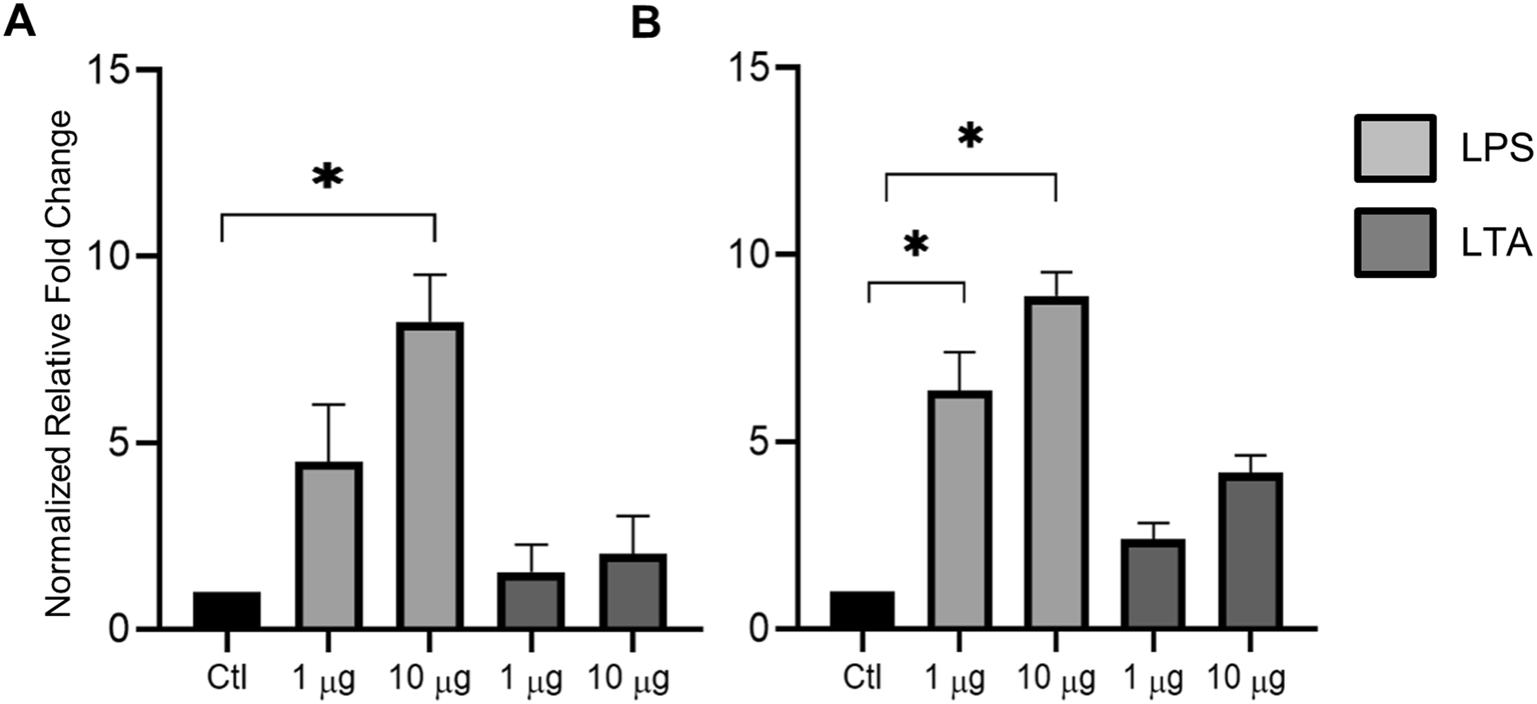
**Bacterial components induce inflammatory responses in 2D enteroids.** Confluent 2D enteroids cultured on uncoated plastic wells were treated on day 5 for 6 h with LTA from *Bacillus subtilis* or LPS from *S. enterica* serotype Typhimurium (1 and 10 μg/mL). Inflammatory responses are assessed by qRT-PCR **A** IL-6 and **B** IL-8 (CXCLi2) mRNA expression shown as fold change relative to control 2D enteroids. Statistical significance calculated in comparison to control cells and shown as *P < 0.05. Data is the median of four independent experiments and 95% CI.

Next, the 2D enteroids were exposed to bacterial endotoxins and epithelial integrity and innate immune responses were assessed. LPS from *S.* Typhimurium, *S.* Enteritidis and *E. coli* or heat-killed bacteria (Figures 6A–D) were added to Maintenance Media in the apical and basal compartments of the 2D enteroids grown on transwells. HiAPEC and LPS up to 10 μg/mL had no effect on the epithelial barrier based on TEER values. Next, day 5 cultures were treated with LPS from *S. typhimurium* or LTA from *B. subtilis* and IL-6 and IL-8 mRNA expression determined 6 h after treatment. IL-6 and IL-8 mRNA expression significantly increased in a dose-dependent manner, while LTA had a minimal effect (Figure 7). The 2D enteroids demonstrated the ability to produce appropriate innate immune responses when exposed to pathogenic and pro-biotic bacterial products.

### Cryopreservation of 2D enteroids

**Figure 8:**
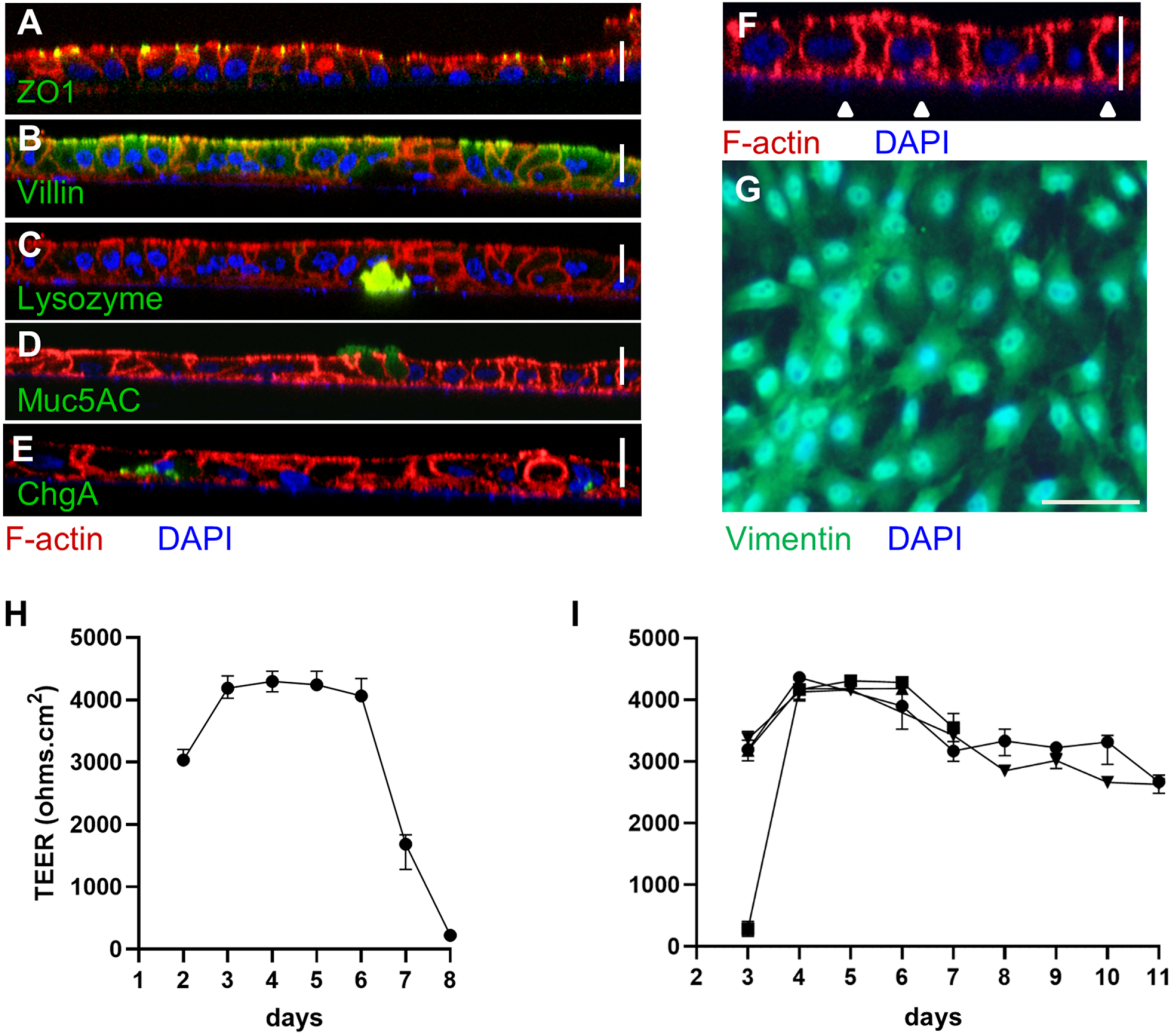
**Resurrection and cellular characterisation of 2D enteroids after cryopreservation.** Immunolocalisation of 2D enteroids grown from cryopreserved intestine cells on Matrigel-coated transwells, on day 4 of culture. Z stack of co-immunolocalisation of F-actin (apical brush border, red) with intestinal epithelial cells (green); **A** ZO1 (Tight Junctions), **B** Villin (enterocytes), **C** Lysozyme (Paneth cells), **D** Muc5AC (Goblet cells) and **E** Chromogranin A (enteroendocrine cells). **F** Magnification of Z-stack of Muc5AC showing DAPI stained nuclei in basal cells (white arrow heads). Scale bars: 15 µm. **G** Vimentin expression (green) in basal mesenchymal cells in the absence of epithelial cells. Scale bar: 50 µm. Nuclei are stained with DAPI. The data represents three independent experiments. **H**, **I** Cells were thawed and grown as 2D enteroids in transwells; CHIR99021 was added to Maintenance Media for **H** 24 h and **I** 72 h. Data is the median of 3–4 independent experiments and 95% CI.

Since passaging of the 2D enteroids is not yet feasible, a protocol that will allow cryopreservation of single cells was developed. The frozen isolated cells could be successfully thawed and formed polarised 2D enteroids 3 days post-seeding containing all epithelial cell lineages (Figures 8A–E) and with a sub-epithelial mesenchymal cell layer present (Figures 8F and G). Thawed cells cultured with CHIR99021 for the first 24 h had similar TEER readings as freshly isolated cells but TEER levels decreased from day 6 (Figure 8H). When thawed cells were cultured with CHIR99021 for 72 h, an increase in barrier integrity based on maximum TEER measurements was found from day 6 up to day 11 (Figure 8I). Increasing the cell seeding density to 2 × 10^5^ cells/well in the latter experiment enabled to extend the maximum TEER similar to fresh cells grown under the same conditions.

## Discussion

Generating well-characterised ex vivo models of the chicken intestine is an essential advancement in poultry research to study their physiology and disease states, to improve health and minimise the number of animal studies. Recent advances in the generation of 3D chicken enteroids have improved the complexity of the cultured organoids to closer resemble native tissue [25], but 2D chicken intestinal models are still relatively primitive with partial characterisation of epithelial cell types, lacking the multi-cellular interactions found in vivo, and with limited scope for functional studies [17–24, 27]. Here, we describe the generation of a confluent 2D epithelial layer from ED18 embryos with all epithelial cell lineages and a sub-epithelial mesenchymal layer, which mirror the architecture of the intestinal lining in vivo. The 2D enteroids were grown successfully in transwell format, with robust epithelial integrity up to 11 days and demonstrating innate immune responses. The derivation of 2D and 3D intestinal enteroids in mammalian species is commonly from the crypts containing stem cells, in contrast the villi of ED18 embryonic chickens were used for the generation of 2D and 3D enteroids, because crypts are at a rudimentary stage of development. It takes to 48 h post hatch for intestinal invaginations to complete with crypt enlargement and fission continuing over 9 days post-hatch [29]. The protocols for preparation of chicken intestinal epithelial cultures are commonly from late-stage embryonic chicks, and therefore by definition derived from villi [17–24, 27]. A mixed population of disaggregated villi cells when randomly seeded was able to generate a self-organised apical layer containing enterocytes, Goblet cells, Paneth cells, enteroendocrine cells and leukocytes and a sub-epithelial mesenchymal layer.

The intestinal mesenchyme is involved in regulation of the intestinal epithelium [2] and can also act as non-professional immune cells [34] during homeostasis, inflammation, injury and repair. The process of spontaneous self-organisation into segregated cell layers from pools of multiple cell types has been demonstrated in other culture systems, for example, formation of models of the skin [35]. The major subsets of mesenchymal cells in the villi are fibroblasts, myofibroblasts and pericytes, and each subset can express one or more markers, vimentin, desmin and α-smooth muscle actin [2, 34, 36], which were all identified in the 2D enteroids. The overlapping nature of marker gene expression in intestinal mesenchymal cells means in the absence of co-localisation studies, cell types in this study are not specified. Mesenchymal cells are often included in intestinal epithelial cultures to support their long-term maintenance [11] and also to generate more organotypic cultures [37]. Long-term cultures were not achieved in our study, but the maximum culture duration of the 2D enteroids (11 days) is not prohibitive to most experiments investigating intestinal permeability and integrity.

The growth factor requirements for maintaining mammalian 3D organoids and intestinal monolayer cultures have been well defined, and consist of Epidermal Growth Factor, Noggin, and R-Spondin (ENR) [4, 9, 12, 20]. The culture conditions of the chicken 2D enteroids were established using a modified ENR growth factor cocktail previously used in mouse intestinal monolayer cultures [12]. In the early culture period additional factors were added including GSK3β inhibitor, which acts as a Wnt activator to support stem cells, proliferative cells and Paneth cells [12, 22, 38] and Rock inhibitor to prevent anoiksis that occurs in dissociated intestine cells ex vivo [39, 40].

The generation of 2D intestinal cultures with a tight epithelium has not previously been reported in chicken [17–24, 27]. Most recently, confluent cultures of primary chicken duodenal epithelial cells were described [20]. These cultures survive until day 12 with tight junctions and a low TEER value of 64 Ω·cm^2^, which is in line with some other intestinal cell models but does not indicate a tight barrier [41]. For comparison, in chicken, TEER values of greater than 10 000 Ω·cm^2^ have been measured in duodenum explants [42]. In the 2D enteroids, lack of paracellular flux of 4 kDa FITC-dextran, robust TEER values (~ 4000 Ω·cm^2^) and immunolocalisation of ZO1, to the apical surface of the epithelium demonstrated the development of a tight functional epithelial barrier.

As a proof-of-concept, the epithelial barrier of the 2D enteroids was positively and negatively manipulated by CHIR99021, Ca^2+^ and NaB. CHIR99021 was added to the early 2D enteroid cultures to support cell growth and might be expected to play a similar role when added to the cultures for longer [12, 22, 38]. An optimal Ca^2+^ concentration is important for tight junction assembly and stabilisation and depletion of Ca^2+^ in cultures of differentiated intestinal cells causes a decrease in epithelial integrity [43, 44] as demonstrated in our leaky gut model. The role of Ca^2+^ concentration in the 2D enteroids and leaky gut model is likely to be through support of intercellular junctions or forming de novo junctions, to maintain and restore epithelial integrity as in other intestinal cell models [43, 44]. Butyrate is used as a feed supplement and alternative to antibiotics and has several roles in the chicken intestine, including support of immune function [45, 46] and possible regulation of tight junction assembly as has been found in intestinal cell lines [47, 48]. The protective and supportive effect of NaB on epithelial integrity found in the 2D enteroids and leaky gut model is consistent with previous reports of NaB able to increase intracellular calcium, activation of the AMPK-activated protein kinase pathway and synthesis of tight junctions in intestine cells [44, 47, 48]. Thus, the chicken 2D enteroids represent a functional model for investigating factors, which regulate epithelial integrity.

Pathogen infection in the intestine can cause epithelial apoptosis, release of inflammatory cytokines, inflammation and decrease in tight junction proteins [1]. Bacterial endotoxins can disrupt intestinal barrier function in vivo in mouse [49] and chickens [50, 51]. When the 2D enteroids were cultured in the presence of LPS or killed APEC, no effect on epithelial integrity was found. LPS alone has been shown previously to not always be sufficient to cause a significant effect on epithelial integrity [52, 53] and the necessity to co-culture with peripheral blood mononuclear cells for LPS to disrupt the epithelium has been reported [54, 55]. To protect epithelial integrity, the intestinal epithelium maintains some hypo-reactivity to microbial ligands as it is constantly exposed to the microbiome. Further, the usual invasive activity of enteropathogenic *E. coli* and *Salmonella* through the type III secretion system [56, 57] may be important to target the actin cytoskeleton and alter membrane integrity. In future studies, the use of invasive bacteria or a co-culture model to investigate the effects of pathogens on epithelial integrity will be tested.

Innate immune responses are critical in the first week post-hatch [45, 58]. Upregulation of inflammatory cytokines, IL-6 and IL-8 mRNA expression found in the 2D enteroids exposed to LPS from *S. Typhimurium* is also previously reported in in young chickens [59, 60] and intestinal epithelial cells [18, 61]. IL-6 and IL-8 may alter epithelial integrity however, it was suggested that their expression alone may not be sufficient to induce significant changes in intestinal epithelial monolayers [55]. Conversely, multiple strains of probiotic *B. subtilis* are used as a safe alternative to antibiotics in chickens, and to promote growth, immunity, epithelial integrity and overall gut health [62, 63] and which is consistent with a minimal effect of LTA from *B. subtilis* in the 2D enteroids. Extensive investigation of the innate immune response in the 2D enteroids in response to microorganisms is a topic for future studies.

Overall, we have developed a protocol to generate chicken 2D enteroids which can be used to perform standardised high-throughput studies of intestinal epithelial cell biology and innate immune function, investigating ways to improve and support intestinal health, and which can be extended to other broader applications.

## Abbreviations

2D: 2 Dimensional
3D: 3 Dimensional
APEC: Avian Pathogenic *Escherichia coli*
DMEM: Dulbecco’s modified eagle medium
ED: Embryonic day
EGF: Epidermal growth factor
GADPH: Glyceraldehyde phosphate dehydrogenase
HiAPEC: Heat inactivated Avian Pathogenic *Escherichia coli*
IL-6: Interleukin 6
IL-8/CxCLi2: Interleukin 8
LCM: Low calcium media
LPS: Lipolysaccharide
LTA: Lipoteichoic acid
MOI: Multiplicity of infection
NaB: Sodium butyrate
PBS: Phosphate buffered saline
r28S: Ribosomal 28S
TEER: Trans epithelial electrical resistance
ZO1: Zona occludens 1
